# Bim Expression Influences Choroidal Endothelial Cell Characteristics and Their Response to Therapeutic Intervention

**DOI:** 10.3390/ijms251910254

**Published:** 2024-09-24

**Authors:** Nader Sheibani, Yong-Seok Song, Mitra Farnoodian, Samay Inampudi, Barbara Hanna, Shoujian Wang, Soesiawati R. Darjatmoko, Christine M. Sorenson

**Affiliations:** 1 Department of Ophthalmology and Visual Sciences, University of Wisconsin School of Medicine and Public Health, Madison, WI 53705, USA; nsheibanikar@wisc.edu (N.S.); song224@wisc.edu (Y.-S.S.); mitra.farnoodian@nih.gov (M.F.); shoujianwang@wisc.edu (S.W.); srdarjat@wisc.edu (S.R.D.); 2 Department of Cell and Regenerative Biology, University of Wisconsin School of Medicine and Public Health, Madison, WI 53705, USA; 3 McPherson Eye Research Institute, University of Wisconsin School of Medicine and Public Health, Madison, WI 53705, USA; 4 Department of Pediatrics, University of Wisconsin School of Medicine and Public Health, Madison, WI 53705, USA; inampudi2@wisc.edu (S.I.); hanna.barbara@mayo.edu (B.H.)

**Keywords:** choroidal neovascularization, age-related macular degeneration, VEGF, mononuclear phagocytes, Bcl-2 family, cell death

## Abstract

In the aging population, choroidal vessels grow through the Bruch’s membrane, resulting in a loss of central vision due to choroidal neovascularization (CNV). During active neovascularization, CNV is associated with inappropriate levels of apoptosis in multiple cell types, including choroidal endothelial cells (ChECs). Bim is a pro-apoptotic member of the Bcl-2 family. It is essential for cell apoptosis due to exposure to drugs such as dexamethasone or decreased pro-survival factors, including vascular endothelial growth factor (VEGF). To better elucidate the cell autonomous contribution of Bim expression in the integrity and neovascularization of the choroidal vasculature, we isolated ChECs from wild-type and Bim-deficient (Bim^−/−^) mice. ChECs lacking Bim expression demonstrated increased expression of VEGF, osteopontin, and the inflammatory cytokines Rantes/Ccl5 and IL6. Bim^−/−^ ChECs were more proliferative and demonstrated an increased capacity to undergo capillary morphogenesis. Anti-VEGF had a diminished capacity to disrupt capillary morphogenesis in Bim^−/−^ ChECs. In vivo, utilizing the mouse laser photocoagulation model, anti-VEGF treatment mitigated CNV in wild-type but not Bim^−/−^ mice. We also tested other modalities that are thought to not require the intrinsic death pathway for their function and showed that propranolol, anti-CTGF, and the TSP1-mimetic peptide ABT898 mitigated CNV in mice lacking Bim expression to varying degrees. Thus, in ChECs, Bim expression could impact the effectiveness of treatment modalities that require the intrinsic death pathway to mitigate CNV.

## 1. Introduction

Neovascular age-related macular degeneration (nAMD) occurs when choroidal vessels aberrantly grow through the Bruch’s membrane, leading to a loss of central vision in older adults. Choroidal neovascularization (CNV) is associated with altered choroidal endothelial cells (ChECs), retinal pigment epithelial (RPE) cells, and macrophage apoptosis during active neovascularization [1]. Unfortunately, there is a limited understanding of the consequences that dysregulated apoptosis has on ChEC characteristics and function. The CNV that accompanies nAMD pathogenesis is largely driven by increased vascular endothelial growth factor (VEGF) expression, the major target of current therapies for this disease. Gaining a better understanding of how Bim expression influences ChEC characteristics and function will allow to better tailor treatment modalities where apoptosis is central to their function.

Apoptosis has an important role during development and disease pathogenesis due to the critical role it plays in the homeostasis of most cells. To maintain tissue homeostasis, apoptosis is tightly regulated, with nearly 50–70 million cells in adults undergoing apoptosis daily [2]. Cells can undergo apoptosis through either intrinsic (also known as mitochondrial, stress-induced, or Bcl-2 regulated) or extrinsic (death receptor-induced) pathways. The intrinsic pathway is triggered in many ways; for example, by growth factor depletion, metabolic stress, hypoxia due to lack of blood flow, or cytotoxic agents [3]. Once a death stimulus is encountered, Bcl-2 family members such as Bim, which contains a single Bcl-2 homology (BH) domain (BH3) [4,5], curtail the function of anti-apoptotic family members to facilitate apoptosis. Frequently, disease pathogenesis is associated with decreased or enhanced apoptosis that affects the removal of imperfect or harmful cells.

Bim is unique within the death effector wing of the Bcl-2 family because it is the only BH3-domain-only protein that initiates EC apoptosis on its own [6,7]. It is transcriptionally and post-transcriptionally regulated. BIM functions intrinsically to initiate EC death due to serum withdrawal, steroid treatment, and VEGF blockade [6,8,9]. BIM polymorphisms that lack a functional BH3 domain decrease BIM expression, causing dexamethasone and many other cancer treatments to be ineffective [10]. These studies highlight the role played by BIM during the therapeutic removal of cells contributing to disease pathogenesis, most notably lung cancer and leukemias [6,11,12]. In the eye, global or EC or pericyte lack of Bim expression significantly decreases hyaloid and postnatal retinal vascular remodeling [7]. During oxygen-induced ischemic retinopathy (OIR), Bim expression drives retinal vessel obliteration due to exposure to high oxygen and response to decreased VEGF levels [6,7]. Although the role Bim plays in retinal vascular remodeling is emerging, substantially less is known regarding the effect of Bim expression in the choroidal vasculature.

The choroidal vasculature is unique compared to other vascular beds, making it difficult to draw parallels from what we know regarding the role Bim plays in retinal, lung, and kidney ECs to ChECs. Here, we examined not only the unique role Bim expression has in ChECs, but also its impact on the efficacy of anti-VEGF treatment in vivo in the laser photocoagulation model. ChECs lacking Bim expression (Bim^−/−^) displayed aberrant angioregulatory factor expression, including VEGF, thrombospondin-1 (TSP1), TSP2, and Bcl-2, and increased inflammatory mediator expression. ChEC proliferation was increased in the absence of Bim, but apoptosis induced by H_2_O_2_ or staurosporine and cell migration were similar in wild-type (WT) and Bim^−/−^ ChECs. Anti-VEGF treatment of WT ChECs or mice prevented capillary morphogenesis in vitro and CNV in vivo in the laser photocoagulation model. In contrast, anti-VEGF treatment was not effective in these models in the absence of Bim expression. We also tested the efficacy of several treatment modalities not noted to be dependent on the intrinsic death pathway for their efficacy and showed that propranolol, anti-CTGF, and the TSP1-mimetic peptide ABT898 significantly inhibited CNV in Bim^−/−^ and/or Bim^MP^ (mononuclear phagocyte (MP)-targeted) mice.

## 2. Results

### 2.1. Bim^−/−^ ChECs Exhibit a Spindly and Elongated Morphology

**Figure 1:**
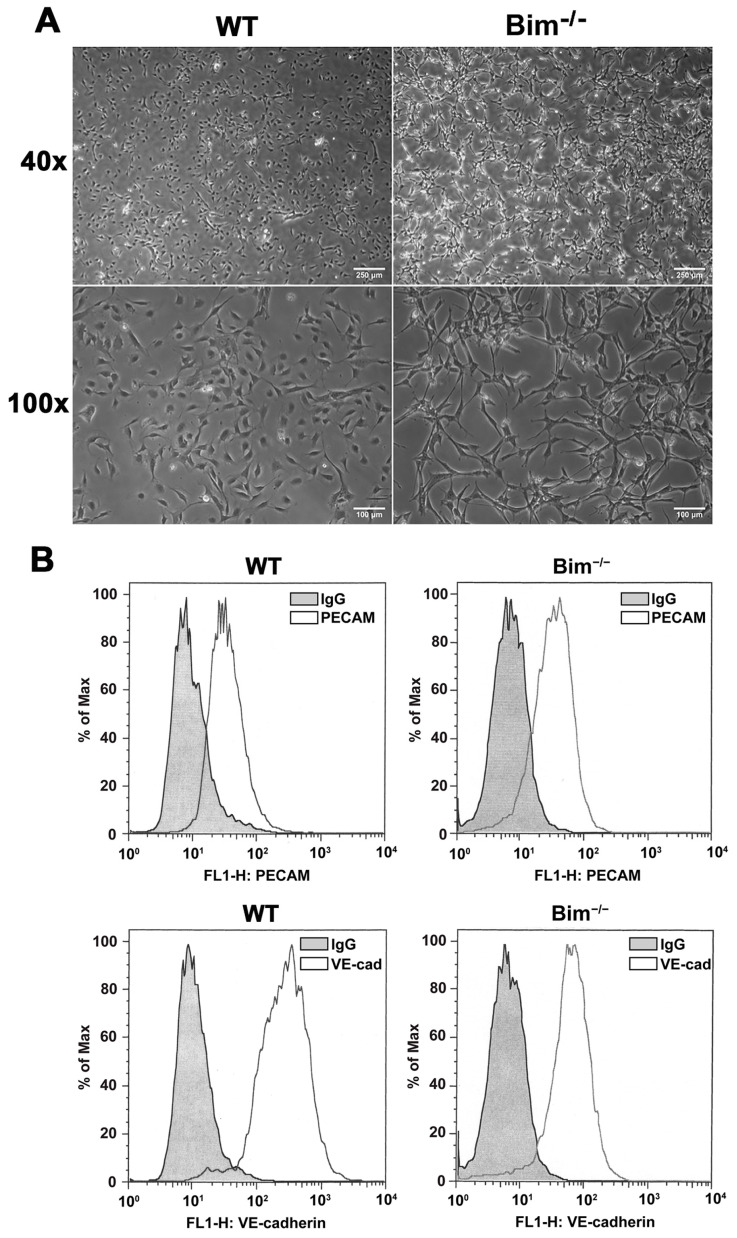
Isolation of wild-type and Bim^−/−^ ChECs. Panel (**A**): ChECs were prepared and cultured on gelatin-coated plates. A phase image was used for morphology comparison. Scale bar = 250 µm (40×) and 100 µm (100×). Please note that Bim^−/−^ ChECs have a more elongated morphology compared to WT ChECs. Panel (**B**): The EC markers VE-cadherin and PECAM-1 were assessed by flow cytometry analysis.

ChEC loss is an early event during AMD pathogenesis [13]. Here, the role Bim expression plays in modulating ChEC characteristics was examined. Morphology was assessed with ChECs plated on gelatin-coated dishes. Figure 1A shows that Bim^−/−^ ChECs appear more elongated compared to their WT counterpart. To ensure the WT and Bim^−/−^ ChECs expressed EC markers, flow cytometry analysis was utilized to assess VE-cadherin and PECAM-1 expression. Figure 1B demonstrates that VE-cadherin and PECAM-1 are expressed in both WT and Bim^−/−^ ChECs, comparable to our previously published reports [14].

### 2.2. Increased VEGF and Inflammatory Mediators in the Absence of Bim

**Figure 2:**
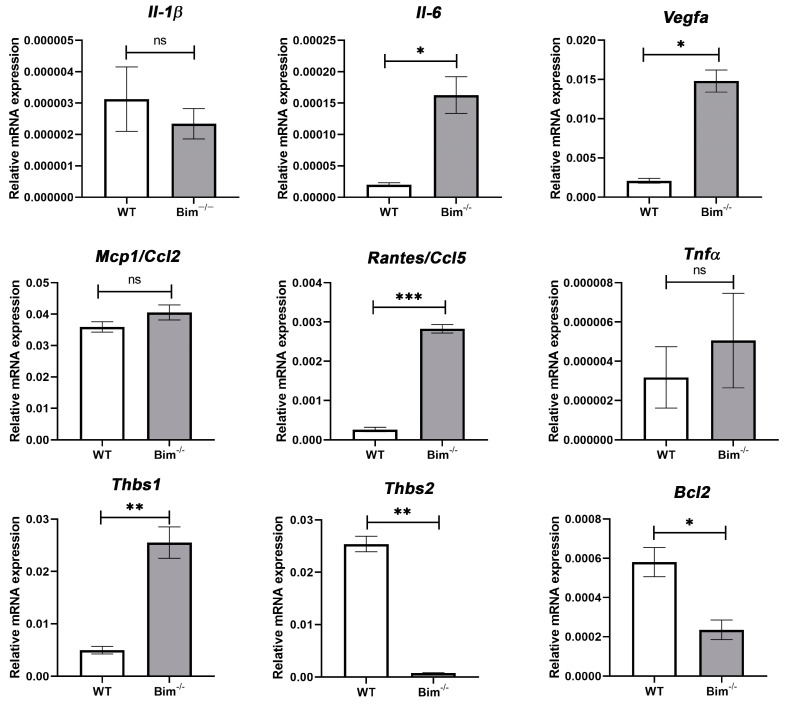
Bim deficiency results in increased VEGF levels. Expression of VEGF, TSP1, TSP2, Bcl-2, and inflammatory mediators was assessed by qPCR analysis in WT and Bim^−/−^ ChECs. These experiments were repeated three times with two different ChEC isolations with similar results. A significant increase in the levels of Il-6, Rantes/Ccl5, VEGF, and TSP1, while a decrease in TSP2 and Bcl-2 levels was observed (* *p*< 0.05, ** *p* < 0.01, *** *p*< 0.001; Student’s unpaired *t*-test (2-tailed) was utilized). RpL13A, 60S ribosomal protein L13a. ns, not significant.

**Figure 3:**
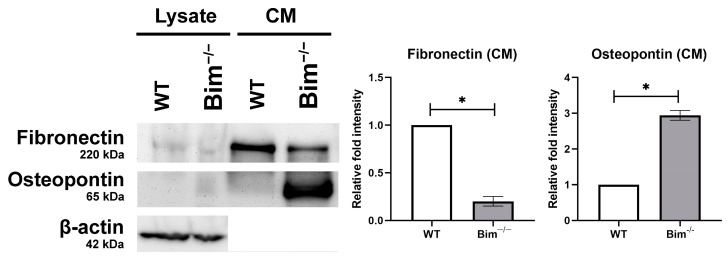
Changes in the expression of osteopontin and fibronectin. Conditioned medium (CM) and cell lysates (25 µg) were analyzed by Western blot analysis. Osteopontin and fibronectin levels, secreted and cell-associated, were assessed in WT and Bim^−/−^ ChECs. β-actin expression was analyzed as a loading control for cell lysates. These experiments were repeated with two different cell isolations in triplicate (* *p*< 0.05, Student’s unpaired *t*-test (2-tailed) was utilized).

**Figure 4:**
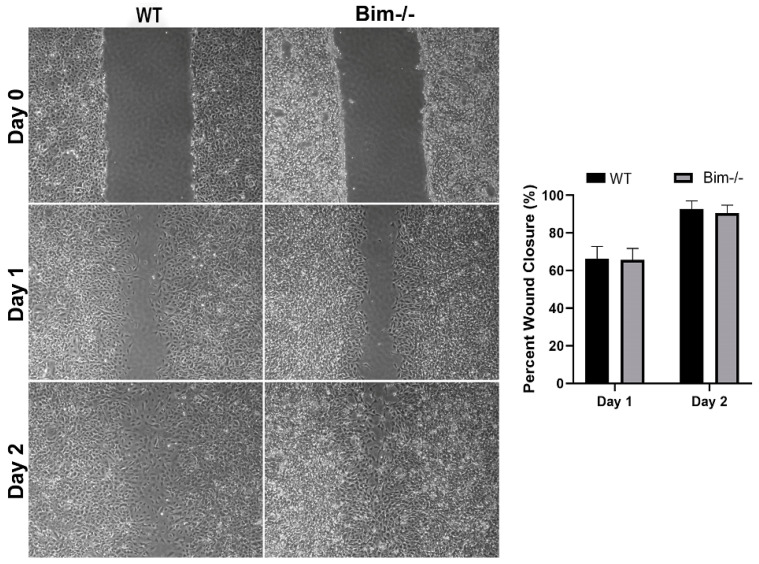
Bim expression does not affect ChEC migration. Migration of WT and Bim^−/−^ ChECs was assessed using a scratch wound assay. Wound closure was assessed at 0, 1, and 2 days from phase images. Both WT and Bim^−/−^ ChECs demonstrated similar migration in this assay. Experiments were repeated three times with two different ChEC isolations with similar results (no significant difference; Student’s unpaired *t*-test (2-tailed) was utilized).

VEGF signaling increases cell survival, proliferation, and migration, all of which drive neovascularization. Thrombospondin (TSP) and Bcl-2 family members influence VEGF signaling and CNV [15,16]. Although VEGF signaling pathways have been well studied in retinal ECs, substantially less is known regarding these signaling pathways in ChECs. Given that the choroidal vasculature is unique compared to other vascular beds, including the retina, the impact of Bim expression on VEGF and inflammatory mediator expression and migration in ChECs was assessed. Bim^−/−^ ChECs expressed significantly more VEGF and TSP1 while TSP2 and Bcl-2 expression decreased compared with WT ChECs (Figure 2). Bim^−/−^ ChECs had significantly increased inflammatory mediator expression, namely Rantes/Ccl5 and Il-6, compared with WT ChECs (Figure 2), while Il-1β, Mcp1/Ccl2, and TNF-α levels were similar between these ChECs (Figure 2). Bim^−/−^ ChECs also produced significantly increased levels of osteopontin, but decreased levels of fibronectin compared with WT cells (Figure 3). Even though VEGF expression significantly increased in Bim^−/−^ cells, ChEC migration was similar in WT and Bim^−/−^ ChECs (Figure 4).

**Figure 5:**
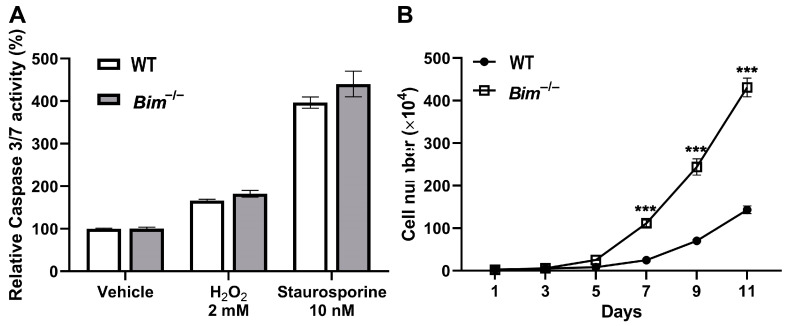
Increased proliferation in Bim^−/−^ ChECs. In Panel (**A**), WT and Bim^−/−^ ChECs were incubated with vehicle, H_2_O_2_ (2 mM), or staurosporine (10 nM) for 24 h. A Caspase 3/7 glo assay was used to assess the levels of apoptotic cells. The growth rate of WT (•) and Bim^−/−^ (□) ChECs (Panel (**B**)) were counted for 11 days. Similar results were observed when these experiments were repeated with two different isolations of ChECs (*** *p*< 0.001; Student’s unpaired *t*-test (2-tailed) was utilized).

Next, the levels of apoptosis and proliferation were assessed in these cells. The basal level and challenged level of apoptosis with apoptotic stimuli (H_2_O_2_ or staurosporine) were determined by measuring caspase 3/7 activation. Figure 5A shows that both WT and Bim^−/−^ ChECs had similar basal and challenged levels of apoptosis (Figure 5A). Proliferation was assessed by counting cell numbers. Figure 5B shows that Bim^−/−^ ChECs were significantly more proliferative compared with WT cells. Thus, Bim^−/−^ ChECs demonstrated increased VEGF, TSP1, and inflammatory mediator expression and proliferation.

### 2.3. ChEC Capillary Morphogenesis, Bim, and Anti-VEGF

**Figure 6:**
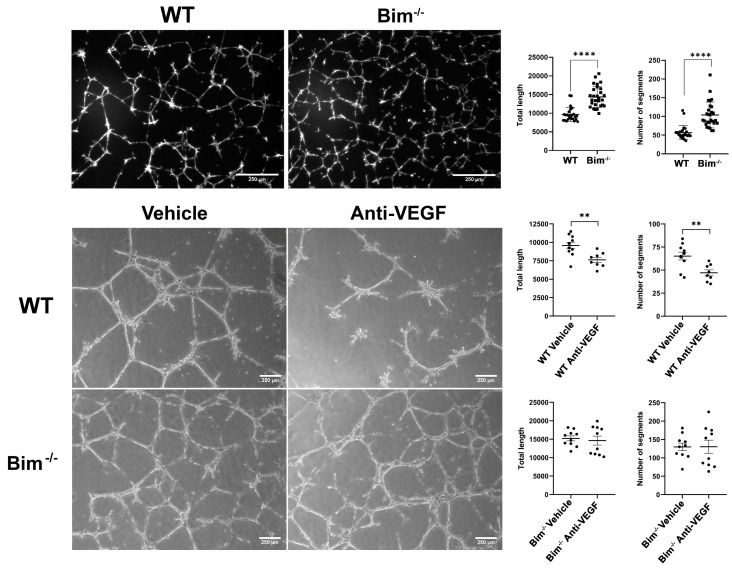
Increased capillary morphogenesis in Bim^−/−^ ChECs. In the upper panel, WT and Bim^−/−^ ChECs were plated on Matrigel; 18 h later, photographs were taken in a digital format. The mean number of segments and total length were determined. In the lower panel, anti-VEGF (1 µg) or vehicle was added to WT and Bim^−/−^ ChECs after overnight plating on Matrigel. Photomicrographs were taken the following day. Similar results were observed with two different isolations of ChECs. The number of segments and total segment length were quantified using the Angiogenesis Analyzer plug-in for ImageJ. Scale bar = 250 µm (** *p*< 0.01, **** *p*< 0.0001; Student’s unpaired *t*-test (2-tailed) was utilized).

CNV relies on ChECs’ ability to form capillary networks. Here, the capacity of WT and Bim^−/−^ ChECs to form capillary networks was assessed. Bim^−/−^ ChECs formed significantly more segments and had increased total capillary length in Matrigel compared with their WT counterpart (Figure 6; top panel). Given the importance of VEGF for capillary network formation, we next assessed the ability of anti-VEGF to disrupt capillary morphogenesis in the presence and absence of Bim expression. Anti-VEGF significantly decreased the total length and segment number of WT ChECs but not Bim^−/−^ ChECs (Figure 6; bottom panel). Thus, a lack of Bim expression hinders the inhibition of capillary morphogenesis by anti-VEGF.

### 2.4. Bim Expression and Efficacy of CNV Treatment Modalities

**Figure 7:**
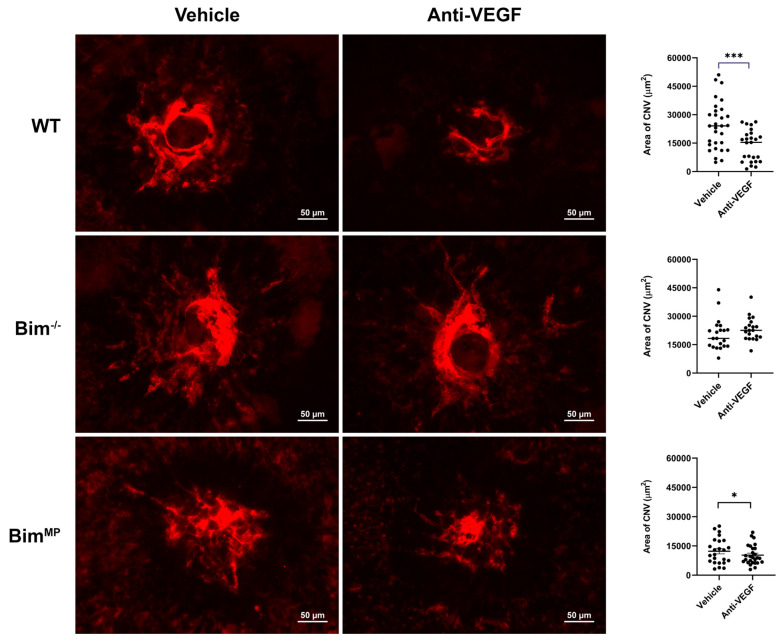
Anti-VEGF decreases CNV in WT but not in Bim^−/−^ mice. Male and female WT, Bim^−/−^, and Bim^MP^ mice subjected to CNV. Mice received intravitreal injection (1 µL) of vehicle (DMSO) or anti-VEGF (1 µL; 25 ng/µL per eye) the day of laser photocoagulation and 7 days later. Two weeks after photocoagulation, the RPE/choroid was wholemount-stained with anti-ICAM-2 and imaged. The total area (in µm^2^) was measured using ImageJ software, version 1.52j (right panel). Each dot on the plot represents one laser spot. Scale bar = 50 µm. All experiments were repeated with at least 8 mice (* *p*< 0.05, *** *p*< 0.001; ANOVA was utilized).

Anti-VEGF therapy is the standard treatment for nAMD patients. Since our in vitro data showed that the ability of anti-VEGF to inhibit ChEC capillary morphogenesis relied on Bim expression, we next determined whether the efficacy of anti-VEGF, in vivo, in the mouse CNV model, required Bim expression. WT mice or those lacking Bim expression globally or in MP (Bim^MP^; generated in the laboratory [17]) were subjected to laser photocoagulation. CNV was assessed 2 weeks later by ICAM-2 staining, a marker of ECs. Anti-VEGF treatment reduced CNV in WT but not in Bim^−/−^ mice (Figure 7). Anti-VEGF treatment also significantly decreased CNV in Bim^MP^ mice. Thus, in vivo as in in vitro, the global deletion of Bim decreased the efficacy of anti-VEGF.

**Figure 8:**
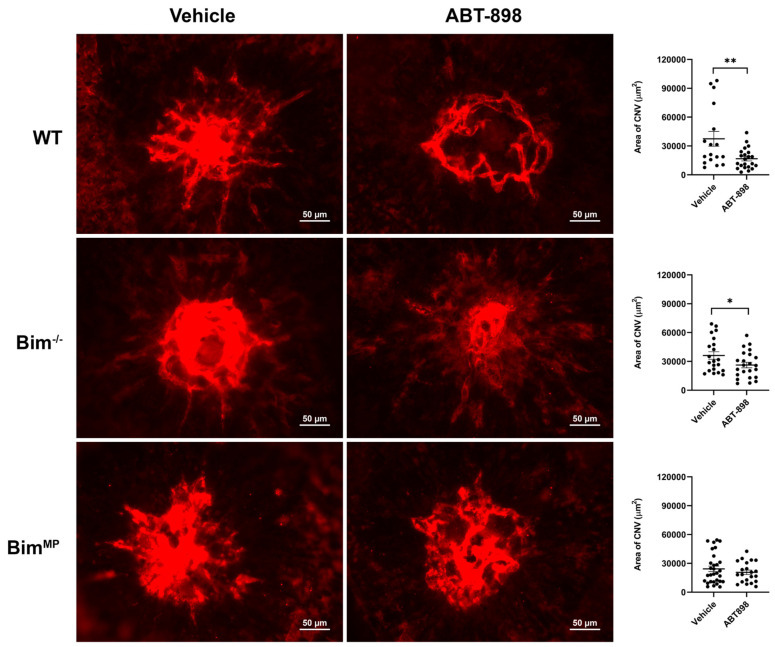
ABT 898 was not effective in reducing CNV in Bim^MP^ mice. Male and female WT, Bim^−/−^, and Bim^MP^ mice underwent laser photocoagulation. Mice received intravitreal injection (1 µL) of either vehicle (BSS) or ABT898 (1 µL; 4 µg/µL per eye) the day of laser photocoagulation and 7 days later. Two weeks after photocoagulation, the RPE/choroid was wholemount-stained with anti-ICAM-2 and imaged. The total area (in µm^2^) was measured using ImageJ software (right panel). Each dot on the plot represents one laser spot. Scale bar = 50 µm. All experiments were repeated with at least 8 mice (* *p*< 0.05, ** *p*< 0.01; ANOVA was utilized).

**Figure 9:**
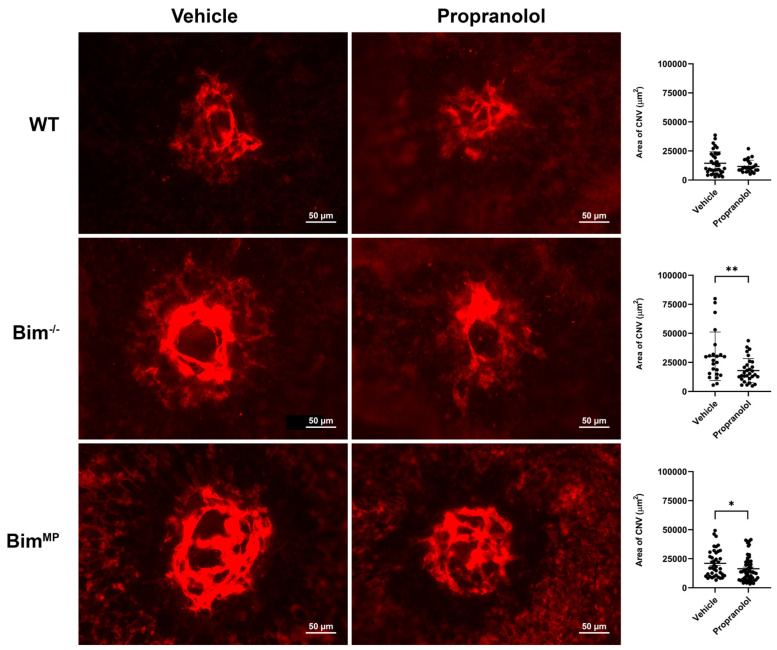
Propranolol effectively reduced CNV in Bim^−/−^ mice. Female WT, Bim^−/−^, and Bim^MP^ mice underwent laser photocoagulation-induced rupture of Bruch’s membrane. Mice received an intraperitoneal injection of vehicle (200 µL PBS) or propranolol (500 µg/200 µL) daily, -3 through 14 days. Two weeks after photocoagulation, the RPE/choroid was wholemount-stained with anti-ICAM-2 and imaged. The total area (in µm^2^) was determined using ImageJ software (right panel). Each dot on the plot represents one laser spot. Scale bar = 50 µm. All experiments were repeated with at least 8 mice (* *p*< 0.05, ** *p*< 0.01; ANOVA was utilized).

**Figure 10:**
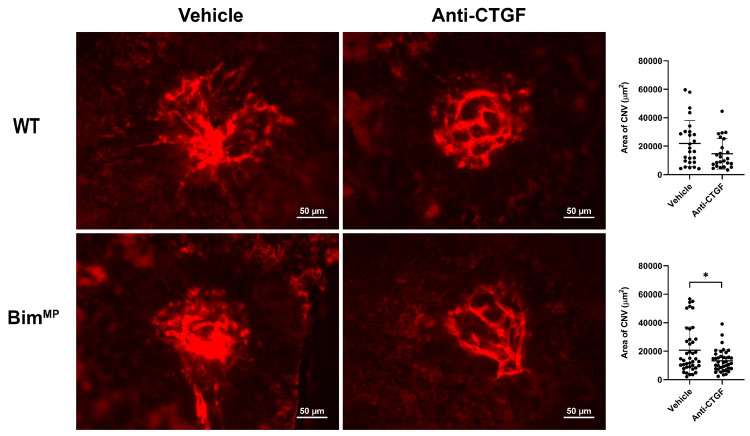
Anti-CTGF reduces CNV in Bim^MP^ mice. Male and female WT and Bim^MP^ mice received intravitreal injection (1 µL) of either vehicle (PBS) or anti-CTGF (1 µL; 50 ng/µL) the day of laser photocoagulation and 7 days later. Two weeks after photocoagulation, the RPE/choroid was wholemount-stained with anti-ICAM-2 and imaged. The total area (in µm^2^) was determined using Image J software (right panel). Each dot on the plot represents one laser spot. Scale bar = 50 µm. All experiments were repeated with at least 8 mice (* *p* < 0.05; Student’s unpaired *t*-test (2-tailed) was utilized).

Next, modalities in which the intrinsic death pathway is not essential for their efficacy were examined using a laser photocoagulation model. These modalities included the TSP1-mimetic peptide ABT898, propranolol, and anti-CTGF (Figure 8, Figure 9 and Figure 10). ABT898 is an octapeptide TSP1-mimetic with increased stability and a longer half-life than its predecessor [18,19]. The treatment of WT mice with ABT898 decreased the level of CNV compared to vehicle-treated mice (Figure 8). Bim^−/−^ mice had a less robust, yet significant, reduction in CNV with ABT898 treatment compared to WT mice. ABT898 treatment of Bim^MP^ mice did not demonstrate a significant reduction in CNV (Figure 8). The β-adrenergic receptor antagonist propranolol reportedly only effectively mitigates CNV in female mice [20,21]. Here, the efficacy of propranolol in female mice, subjected to laser photocoagulation, was assessed in the presence and absence of Bim. WT female mice demonstrated a downward trend in CNV following propranolol treatment which did not reach significance, a similar but not as robust decline as that seen in previous studies by others [20,21]. However, in Bim^−/−^ and Bim^MP^ female mice, the propranolol treatment significantly decreased CNV following laser photocoagulation (Figure 9). CTGF regulates fibrotic and angiogenic factors as a response to a wide range of stimuli [22,23]. Figure 10 demonstrates a significant inhibition of CNV in Bim^MP^ mice with anti-CTGF treatment. Thus, ABT898, propranolol, and anti-CTGF, unlike anti-VEGF, mitigated CNV in mice lacking Bim expression to varying degrees.

## 3. Discussion

The choriocapillaris has an important role in maintaining vision [24]. The choroidal circulation maintains the photoreceptor and outer retinal health. Disruption of the choroidal circulation impacts the pathogenesis of vision loss by such eye diseases as AMD. AMD is the predominant cause of vision impairment in the aging population. The neovascular form accounts for practically 90% of severe vision loss among nAMD patients, even though nAMD is less predominant than dry AMD. Inflammation is a driving force in nAMD, with MP acting in a detrimental fashion by accumulating in the subretinal space enhancing CNV and RPE and photoreceptor degeneration [25,26]. Although VEGF expression is needed for maintaining the RPE, too much expression contributes to CNV and vessel leakiness. Thus, VEGF is targeted as the main anti-angiogenic therapy, with some nAMD patients observing increased vision acuity. 

A significant portion of nAMD patients (~30–50%) have an incomplete response to anti-VEGF treatment [27,28]. In addition, about 20–25% of patients receiving anti-VEGF treatment exhibit RPE atrophy, further showing the need for VEGF to maintain tissue homeostasis [29,30]. Although both vascular cells and MP contribute to the VEGF pool, our recent studies showed that MP turnover limits fibrosis following laser photocoagulation [17]. Anti-VEGF therapy decreases EC survival and pathological vessel growth during CNV but does not impact accompanying fibrosis. Here, we show that ChECs, like other vascular and epithelial cells lacking Bim expression, express significantly more VEGF than their WT counterparts [31,32,33]. ChECs, like their retinal and lung equivalents, also demonstrate increased proliferation in the absence of Bim. However, unlike any other Bim-deficient cells we have examined, Bim^−/−^ ChECs were not protected from apoptosis by either staurosporine or H_2_O_2_ [31,32,33]. We had previously reasoned that Bim^−/−^ cells were resistant to apoptosis, due to increased VEGF expression [31,32,33]. This does not appear to be the complete story, as Bim^−/−^ ChECs showed significantly increased VEGF expression but underwent apoptosis at the same rate as WT ChECs when challenged. Perhaps in ChECs a lack of Bim expression increases VEGF expression, driving proliferation and inflammation, but the ratio of Bim to Bcl-2 defines the rate of apoptosis. Bcl-2 expression is decreased in Bim^−/−^ ChECs, unlike many other Bim^−/−^ cells where Bcl-2 expression remains constant, tipping the ratio in favor of Bcl-2 and survival in the absence of Bim.

**Figure 11:**
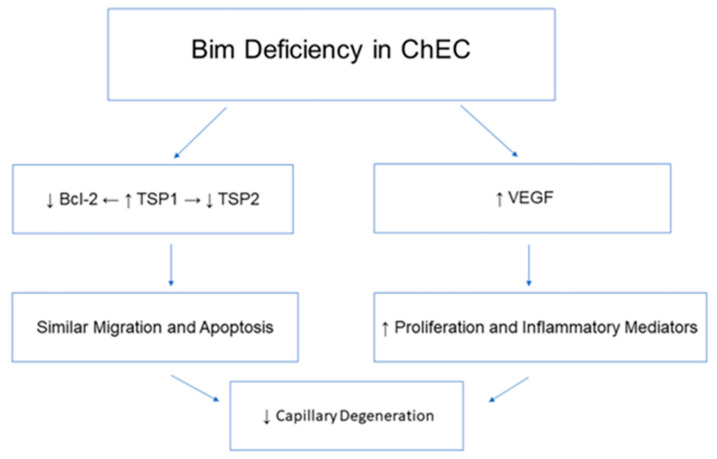
Summary of lack of Bim expression in ChECs. The diagram illustrates the changes that occur in ChECs in the absence of Bim expression. It summarizes data shown in Figure 1, Figure 2, Figure 3, Figure 4, Figure 5 and Figure 6.

Although choroidal endothelium dysfunction contributes to nAMD, the formation of the mature ChEC network has not been as extensively studied. ChECs are fenestrated, aiding in rapid nutrient transport to the RPE, which responds to growth factors including VEGF, FGF2, and IGF-1. The mechanisms underlying these characteristics are not well defined. This unfortunately leaves gaps in our understanding as to the most effective method to treat diseases of the choriocapillaris. Bim^−/−^ ChECs undergo increased stable capillary branching compared to their WT counterpart, even though EC migration was similar. Our previous studies of Bim^−/−^ retinal and lung ECs showed enhanced migration compared to their WT counterpart, which was negated when Bcl-2 was also absent [32,33]. The significant decrease in Bcl-2 expression in Bim^−/−^ ChECs, perhaps due to its suppression by increased TSP1 expression, likely tempers ChEC migration in the absence of Bim. We also noted a significant decrease in TSP2 expression, consistent with a lower level of oxidative stress and increased TSP1 levels. Thus, changes resulting from a loss of Bim expression appears to impact the unique ChEC environment differently than other vascular beds, including that of the retina (summarized in Figure 11).

In nAMD patients that receive anti-VEGF therapy, VEGF levels should be lowered to a level that prevents vessel permeability and pathologic neovascularization but not to such low levels that it results in RPE loss. Since the effectiveness of a VEGF blockade is reported to rely upon a targeted cell’s Bim expression, at least in cancer patients [6], we initially investigated its impact on ChECs with and without Bim expression. This is of particular interest since the intent of Bim expression in this context is to induce EC apoptosis squelching neovascularization. Here, we show that while anti-VEGF incubation decreased the total capillary length and segment numbers in WT ChECs, it had no significant impact on Bim^−/−^ ChECs. These data support a role in the choroid for Bim expression influencing the efficacy of anti-VEGF, as was previously reported in cancer therapy [6].

Anti-VEGF is the foremost treatment for nAMD. We extended our in vitro studies to assess the efficacy of anti-VEGF CNV treatment using a mouse model of laser-induced photocoagulation. Comparable to what we observed in vitro, anti-VEGF treatment in WT mice decreased CNV, while mice lacking Bim, either globally or in MP, did not demonstrate a significant decrease in CNV. Utilizing CNV treatments that, to our knowledge, did not rely on Bim expression or utilize the intrinsic cell death pathway, we show a varying ability to moderate CNV. ABT898 is a TSP1-mimetic peptide that effectively and reproducibly decreases CNV in WT mice. In Bim^−/−^ mice, we observed a more modest decline in CNV with ABT898 treatment while Bim^MP^ mice did not show a significant impact with treatment. Perhaps this is explained by the inter-relationships between VEGF, TSP1, and Bcl-2 family members. However, propranolol and anti-CTGF did effectively reduce CNV in Bim^MP^ mice. Retrospective studies show that beta-blocker treatment correlates with a decreased need for anti-VEGF administration in nAMD patients [34]. Thus, utilizing treatment modalities that exert their effects through divergent pathway(s) may be an effective way to sidestep an incomplete response to treatment.

## 4. Materials and Methods

### 4.1. Cell Lines and Experimental Animals

The experiments delineated here were performed in accordance with the Association for Research in Vision and Ophthalmology for utilizing animals in Ophthalmic and Vision Research and were approved by the Institutional Animal Care and Use Committee of the University of Wisconsin School of Medicine and Public Health. Wild-type and Bim^−/−^ mice, generated from Bim^+/−^ breeder pairs, were screened as previously described (Stock #004525; Jackson Laboratory, Bar Harbor, ME, USA) [7]. The conditional Bim mice (Bim^Flox/Flox^) were obtained from Dr. Andreas Strasser and Dr. Phillipe Bouillet, and were healthy and extensively characterized [17,35]. Bim^Flox/Flox^ mice were crossed with Lyz2-Cre (Stock #004781; Jackson Laboratory, Bar Harbor, ME, USA) mice as we previously described to generate Bim^Flox/Flox^ mice that also expressed Lyz2-Cre (mononuclear phagocyte (MP)-targeted mice are called Bim^MP^). Genotyping was performed as previously described [17]. 

Mouse choroid from wild-type and Bim^−/−^ Immortomice was harvested and rinsed with serum-free Dulbecco’s Modified Eagles’s Medium (DMEM; D-5523, Sigma, St Louis, MO, USA), and ChECs were isolated [14]. ChECs were maintained at 33 °C with 5% CO_2_ in DMEM containing 10% fetal bovine serum (FBS; 26140-079, ThermoFisher Scientific, Carlsbad, CA, USA), 2 mM L-glutamine, 2 mM sodium pyruvate, 20 mM HEPES, 1% nonessential amino acids, 100 µg/mL streptomycin, 100 U/mL penicillin, 30 µg/mL of EC growth supplement from bovine neural tissue (#E2759; Sigma, St. Louis, MO, USA), 55 U/mL heparin (#H3149-250KU; Sigma, St. Louis, MO, USA), and 44 U/mL interferon-γ (INF-γ; #483MI100, R&D, Minneapolis, MN, USA) on 60 mm tissue culture dishes coated with 1% gelatin (#G1890, Sigma, St. Louis, MO, USA). Cells were used for experiments (except scratch wound migration) at 70–80% confluence. For the studies delineated below, at least two isolations of ChECs were utilized and all experiments were repeated at least once (n ≥ 4). For experiments, ChECs were grown in medium without IFN-γ in a 37 °C tissue culture incubator for at least 48 h to remove any contribution from the endogenous expression of the temperature-sensitive large T antigen [36]. 

### 4.2. FACS Analysis

FACS analysis for EC markers for ChECs was performed using a 60 mm dish of ChECs for each condition. The dishes were rinsed with phosphate-buffered saline (PBS) containing 0.04% EDTA and then the cells were removed from the dish with cell dissociation solution (Tris-buffered saline; TBS- 20 mM Tris-HCl and 150 mM NaCl; pH7.6 containing 2 mM EDTA with 0.05% BSA). The cells were rinsed with TBS, placed on ice, and blocked in TBS containing 1% goat serum. Next, the cells were incubated with anti-VE cadherin (#ALX-210-232-C100; Enzo Life Sciences, Farmingdale, NY, USA) or anti-PECAM-1 (#550274; BD Biosciences, Chicago, IL, USA). The cell suspension was washed twice and incubated on ice for 30 min with the appropriate secondary antibody diluted in TBS containing 1% BSA (FITC-conjugated; Jackson ImmunoResearch, West Grove, PA, USA). Following the staining protocol, the cells were washed and resuspended in TBS containing 1% BSA for FACScan analysis (BD Biosciences, Chicago, IL, USA).

### 4.3. Proliferation and Apoptosis

ChEC proliferation was assessed by counting cell numbers every other day for nearly two weeks. ChECs were plated on multiple sets of gelatin-coated 60 mm tissue culture dishes (2 × 10^4^ cells), in triplicate, for each time point. Cell numbers were assessed every other day by trypsinization and manual counting. Cells were given fresh medium on alternate days.

To assess cell death, ChECs were grown on gelatin-coated 96-well plates, in triplicate, overnight in growth medium. The next day, they were subjected to an apoptotic challenge (10 nM Staurosporine (#ALX-380-014-M001; Enzo Life Sciences) or 2 mM H_2_O_2_ (#H1065-100 mL; ThermoFisher, Waltham, MA, USA) for 24 h. Cell viability was assessed using a caspase 3/7 Glo kit (#G8091; Promega, Madison, WI, USA) as recommended by the supplier.

### 4.4. Western Blot Analysis

ChECs (6 × 10^5^) were plated on gelatin-coated 60 mm tissue culture dishes and grown to ~90% confluence. The dishes were then rinsed with serum-free DMEM, and the cells maintained in serum-free DMEM for the next 2 days. The conditioned medium was clarified, and the cells lysed in 100 µL of lysis buffer (50 mM HEPES, pH7.5, 100 mM NaCl, 0.1 mM EDTA, 1 mM CaCl_2_, 1 mM MgCl_2_, 1% Triton X-100, 1% NP40), and a protease inhibitor cocktail (Roche Biochemical, Mannheim, Germany) which was analyzed using 4–20% Tris-glycine gel (ThermoFisher, Waltham, MA, USA) by SDS-PAGE and the proteins were transferred to a nitrocellulose membrane. Blocked membranes had the following primary antibodies added: anti-fibronectin (#F3648; Sigma) and anti-osteopontin (AF808; R&D Systems, Minneapolis, MN, USA). The membranes were then washed and incubated with a suitable Peroxidase AffiniPure secondary antibody (1:5000 Jackson ImmunoResearch, West Grove, PA, USA). The membranes were developed using Amersham ECL (GE, Pittsburgh, PA, USA), imaged and quantitated using a UVP Biospectrum 810 multispectral imaging system (Upland, CA, USA).

### 4.5. Scratch Wound Assay

ChECs were plated at a density of 10^6^ cells in a gelatin-coated 60 mm tissue culture dish. At confluence, ~2 days later, a wound was made with a 1 mL pipet tip. The dish was then rinsed and growth medium containing 150 ng/mL 5-fluorouracil (#F6627; Sigma, St. Louis, MO, USA) was added to block proliferation. Wound coverage was imaged daily. The original wound size was compared to closure at subsequent days along multiple sites and the distance migrated calculated.

### 4.6. Capillary Morphogenesis Assays

ChECs (10^5^/mL) in serum-free DMEM medium were layered on Matrigel-coated plates (0.5 mL), maintained at 37 °C in a tissue culture incubator (35 mm; #12556000, Fisher Scientific, Waltham, MA, USA), and photographed with a Nikon microscope (ECLIPSE TS100; Nikon, Melville, NY, USA) in a digital format. The number of segments and total segment length from 5 high-power fields (×100) of each dish was quantified and a mean for each condition determined using the Angiogenesis Analyzer plug-in for ImageJ to analyze cellular branched networks [37]. To assess the utility of anti-VEGF to induce regression, 1 µg of anti-VEGF_164_ antibody (#AF-493-SP; R&D Systems, Minneapolis, MN, USA) or vehicle (control IgG) was added once capillary morphogenesis was established. Photomicrographs were taken the subsequent day.

### 4.7. RNA Purification and Real-Time qPCR Analysis

ChECs were lysed in Trizol reagent (#15596018; ThermoFisher, Waltham, MA, USA) and total RNA was extracted using a RNeasy mini kit (#74014; Qiagen, Maryland, CA, USA). For cDNA synthesis with RNA to cDNA EcoDry Premix (#639549; TaKaRa, Mountain View, CA, USA), we used 1 µg of total RNA. The cDNA was diluted 1:10 for the qPCR and samples were run in triplicate for each biological replicate on a Mastercycler Realplex (Eppendorf) with TB-green advantage qPCR premix (#39676; TaKara, Mountain View, CA, USA). The amplification parameters used the primers in Table 1, with standard curves generated from known quantities of each target gene from the linearized plasmid. The linear regression line for DNA (ng) was determined with relative fluorescent units (RFU) at a threshold fluorescence value (Ct). Gene targets were quantified from cell extracts comparing RFU at the Ct to the standard curve. This was then normalized by the simultaneous amplification of the housekeeping RpL13a.

### 4.8. Laser Photocoagulation

For laser photocoagulation, mice of both sexes (10 weeks old) were given anesthesia (ketamine hydrochloride (80 mg/kg) and xylazine (10 mg/kg)). Their pupils were dilated with Tropicamide (1%). To locate positions 9, 12, and 3 o’clock on the posterior pole of each eye, an OcuLight GL diode laser fitted with a slit lamp delivery system (Iridex, Mountain View, CA, USA) was utilized. A handheld cover slip allowed us to view the retina and rupture of the Bruch’s membrane (75 µm spot size, 0.1 sec duration, 120 mW). In some cases, male and female mice were treated with vehicle or anti-VEGF (#AF-493-SP; R&D Systems), anti-CTGF (#500-P252-50UG; PeproTech, Rocky Hill, NJ, USA), or ABT898. Mice received an intravitreal injection (1 µL) of either vehicle (HBSS (anti-VEGF), HBSS (anti-CTGF) HBSS (ABT898)), anti-VEGF (1 µL; 25 ng/µL), anti-CTGF (1 µL; 50 ng/µL), or ABT898 (1 µL; 4 µg/µL) per eye the day of laser photocoagulation and 7 days later. These doses are based on previously published studies that were found to be most efficacious. ABT898 was generously provided by Dr. Jack Henkin (Northwestern University). For studies with propranolol (P0884-1G; Sigma), female mice received an intraperitoneal injection of vehicle (200 µL PBS) or propranolol (500 µg/200 µL) daily, -3 (3 day before laser) through 14 days. Only female mice were utilized for studies with propranolol given previous reports delineating their responsiveness to treatment but not male mice [38].

The choroid-RPE tissue was harvested 2 weeks following laser photocoagulation, fixed in 4% paraformaldehyde (PFA), and incubated with blocking buffer (20% normal goat serum and 5% fetal calf serum in 1x PBS) for 1 h then incubated with anti-collagen I (#ab34710; Abcam, 1:500 in 1x PBS with 20% normal goat serum and 20% fetal calf serum) anti-ICAM-2 (#553326; BD BioSciences, Chicago, IL, USA; 1:500) overnight at 4 °C overnight. ICAM-2 is an endothelial cell marker that is constitutively expressed and used to stain the blood vessels. The appropriate secondary antibody was added (Jackson ImmunoResearch, West Grove, PA, USA; 1:500), digital images were taken with a Zeiss microscope (Zeiss, Chester, VA, USA), and pixel intensities (in µm^2^) were evaluated with ImageJ software (National Institute of Mental Health, Bethesda, MD, USA; https://imagej.net/; accessed on 15 May 2023).

### 4.9. Data Analysis

Differences were evaluated (between 2 groups) with Student’s unpaired t-test (two-tailed). If more than 2 groups were evaluated, we used the one-way ANOVA followed by Tukey’s Multiple Comparison Test using GraphPad Prism 8.0 (GraphPad Software, San Diego, CA, USA). Tukey’s Multiple Comparison Test was used to assess the significant differences between the means of every possible two groups in all experimental groups of three or more. Mean ± SD is shown in the figures. A value of *p* < 0.05 was considered significant.

## Figures and Tables

**Table 1:** Primers used for qPCR analysis.

Gene	ID	Forward 5′ to 3′	Reverse 5′ to 3′
*Bcl-2*	NM_009741.5	*GGAGAGCGTCAACAGGGAGA*	*CAGCCAGGGAAATCAAACAGAG*
*Bim EL*	NM_207680.2	*AGTGTGACAGAGAAGGTGGACAATT*	*GGGATTACCTTGCGGTTCTGT*
*Il1b*	NM_008361	*GTTCCCATTAGACAACTGCACTACA*	*CCGACAGCACGAGGCTTTT*
*Il6*	NM_031168.1	*CAACCACGGCCTTCCCTACT*	*TTGGGAGTGGTATCCTCTGTGA*
*Mcp1*	NM_011333.3	*GTCTGTGCTGACCCCAAGAAG*	*TGGTTCCGATCCAGGTTTTTA*
*Rantes*	NM_013653.3	*GCCCACGTCAAGGAGTATTTCT*	*CAAACACGACTGCAAGATTGGA*
*Tnfa*	NM_013693.2	*ACCGTCAGCCGATTTGCTAT*	*TTGACGGCAGAGAGGAGGTT*
*Thbs1*	NM_011580	*TGGCCAGCGTTGCCA*	*TCTGCAGCACCCCCTGAA*
*Thbs2*	NM_011581.3	*CCCCAAACTGCCAAATTCC*	*TCGTCACAAGCATCTCCGATT*
*Vegfa* *isoforms*	NM_001025257 NM_95200 NM-001025250	*GGAGAGCAGAAGTCCCATGA*	*ACTCCAGGGCTTCATCGTTA*
*Rpl13a*	NM_009438.4	*TCTCAAGGTTGTTCGGCTGAA*	*GCCAGACGCCCCAGGTA*

## Data Availability

All the data supporting the reported results are included in the manuscript.
